# *Lactobacillus plantarum* Enhanced IL-22 Production in Natural Killer (NK) Cells That Protect the Integrity of Intestinal Epithelial Cell Barrier Damaged by Enterotoxigenic *Escherichia coli*

**DOI:** 10.3390/ijms18112409

**Published:** 2017-11-13

**Authors:** Yueqin Qiu, Zongyong Jiang, Shenglan Hu, Li Wang, Xianyong Ma, Xuefen Yang

**Affiliations:** 1 Institute of Animal Science, Guangdong Academy of Agricultural Sciences, Guangzhou 510640, China; qiuyueqin87@126.com (Y.Q.); jiangz38@gmail.com (Z.J.); hushenglan@gdaas.cn (S.H.); wangli1@gdaas.cn (L.W.); maxyw28@163.com (X.M.); 2 State Key Laboratory of Livestock and Poultry Breeding, Guangzhou 510640, China; 3 Key Laboratory of Animal Nutrition and Feed Science in South China, Ministry of Agriculture, Guangzhou 510640, China; 4 Guangdong Key Laboratory of Animal Breeding and Nutrition, Guangzhou 510640, China; 5 Guangdong Public Laboratory of Animal Breeding and Nutrition, Guangzhou 510640, China

**Keywords:** *Lactobacillus plantarum*, *Escherichia coli* K88, NK cells, NCM460 cells, intestinal epithelial barrier, integrity, IL-22

## Abstract

Interleukin (IL)-22-producing Natural Killer (NK) cells protect the gut epithelial cell barrier from pathogens. A strain of probiotics, *Lactobacillus plantarum* (*L. plantarum*, LP), was previously found by our laboratory to significantly improve the mucosal barrier integrity and function of the small intestine in pigs. However, it was unclear whether LP benefited the intestinal mucosal barrier via interactions with the intestinal NK cells. The present study, therefore, was focused on the therapeutic effect of NK cells that were stimulated by LP on attenuating *enterotoxigenic Escherichia coli* (ETEC)-induced the damage to the integrity of the epithelial cell barrier. The results showed that LP can efficiently increase protein levels of the natural cytotoxicity receptor (NCR) family, and the expression levels of IL-22 mRNA and protein in NK cells. Transfer of NK cells stimulated by LP conferred protection against ETEC K88-induced intestinal epithelial barrier damage in NCM460 cells. We found that NK cells stimulated by LP could partially offset the reduction in NCM460 cell monolayers transepithelial electrical resistance (TEER) caused by ETEC K88, and increase ZO-1 and occludin mRNA and protein expressions by ETEC K88-infected NCM460 cells. Furthermore, adding NK cells that were stimulated by LP to ETEC K88-infected NCM460cells, IL-22R1, p-Stat3, and p-Tyk2 expression by NCM460 cells was increased. Mechanistic experiment showed that NK cells stimulated by LP lost the function of maintaining TEER of NCM460 cells challenged with ETEC K88, when polyclonal anti-IL-22 antibody was used to block IL-22 production. Collectively, our results suggested that LP stimulation of NK could enhance IL-22 production, which might be able to provide defense against ETEC-induced damage to the integrity of intestinal epithelial barrier.

## 1. Introduction

The intestinal epithelium barrier plays an important role in separating the internal from the external environment, providing the major physical barrier against the invasion and diffusion of enteropathogenic microorganisms [1]. Pathogens such as *Escherichia coli* (ETEC) can decrease the expression of tight junction proteins, and disrupt the tight junction structures of the mucosal barrier, leading an initial defect of the intestinal barrier function [2,3]. Lodemann and coworkers have demonstrated that ETEC K88 can affect the barrier function of both porcine and human intestinal epithelial cells [4]. A study by Yu and coworkers also showed that ETEC K88 induced damage to the integrity of human Caco-2 cells [5].

In contrast to ETEC, increasing evidence has reported that probiotic bacteria can exert preventive and therapeutic effects in animal models of gastrointestinal disorders [6,7]. *Lactobacillus plantarum* (LP), a strain of probiotics, is commonly found in many fermented foods. Previous work from our laboratory found that LP prevented diarrhea in weanling piglets challenged with ETEC K88 through improving mucosal barrier integrity and function of the small intestine [8]. A study by Liu et al. found that LP was able to protect against dysfunction of the normal human colon cell (NCM460) intestinal epithelial barrier caused by ETEC K88 [9].

NK cells play a critical role in immune response and provide immediate defense against intestinal pathogens [10]. Some studies reported that some strains of probiotics can promote IL-12 [11] and IFN-γ [12] production by NK cells, and enhance the NK activity of peripheral blood mononuclear cells in healthy low-NK individuals and the elderly. However, some studies showed that NK cells also play negative regulatory roles [13]. A study by Satoh-Takayama et al. reported that intestinal microbial flora drove NK cells to produce IL-22 [14], a member of the IL-10-related family, and played an important role in maintaining epithelial cell integrity [15]. Maroof et al. showed that activated NK cells in the spleen can produce IL-10 against chronic *Leishmania* infection [16]. Whether or not NK cells that are stimulated by LP produce IL-22 and IL-10, however, remains to be defined. It was also unclear whether LP benefited intestinal mucosal barrier via interactions with the intestinal NK cells. In this study, we hypothesized that LP could enhance IL-22 expression by NK cells that were able to provide defense against the damage to integrity of intestinal epithelial barrier by ETEC. Thus, the aim of this study was to investigate whether NK cells stimulated by LP were able to protect against intestinal injury induced by ETEC challenge, and the related signaling pathways were investigated.

## 2. Results

### 2.1. Effect of Lactobacillus plantarum on Natural Cytotoxicity Receptors (NCRs) Proteins Level in Natural Killer (NK) Cells

**Figure 1:**
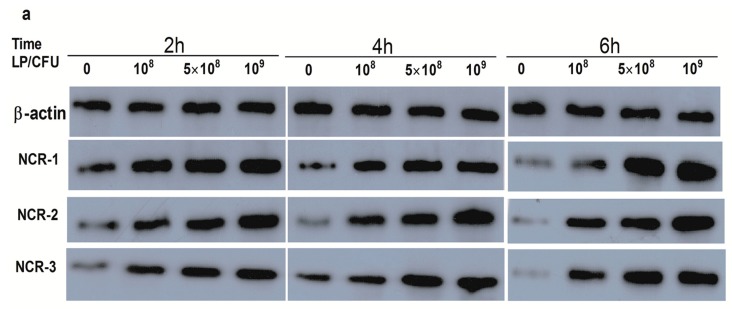

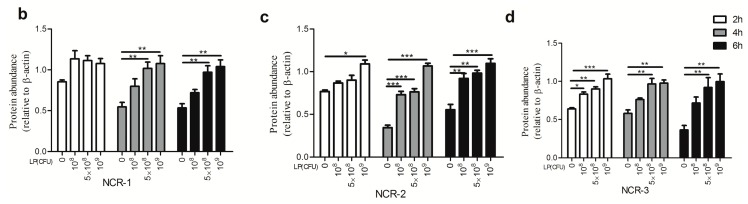
*Lactobacillus plantarum* (LP) increased the expression of natural cytotoxicity receptor (NCRs) protein levels in Natural Killer (NK) cells. NK cells were untreated or treated with *L. plantarum* (10^8^, 5 × 10^8^ or 10^9^ CFU/mL) for 2, 4 or 6 h. Cells were collected and protein abundances were analyzed. (**a**) Western blot analysis of NCR1-3 protein, equal loading was confirmed by stripping immunoblots and re-probing for β-actin; (**b**–**d**) are the ratios of NCR-1, NCR-2, and NCR-3 to β-actin, respectively. All data were obtained from 3 independent experiments. Data are means ± standard error of the means (SEM). * *p* < 0.05, ** *p* < 0.01, *** *p* < 0.001. NCR-1, natural cytotoxicity receptor 1; NCR-2, natural cytotoxicity receptor 2; NCR-3, natural cytotoxicity receptor 3.

Different concentrations of LP increased the protein level of NCR3, but there was no effect of LP on the expression of NCR1, and only a higher concentration of 10^9^ CFU/mL of LP elevated the NCR2 protein level at 2 h (Figure 1b–d). After 4 h and 6 h of incubation with LP (10^8^, 5 × 10^8^ and 10^9^ CFU/mL), expression of NCR2 protein was markedly increased (Figure 1c). The NCR1 and NCR3 protein levels were significantly enhanced by LP (5 × 10^8^ and 10^9^ CFU/mL) at 4 and 6 h (Figure 1b,d).

### 2.2. Effect of Lactobacillus plantarum on Expression of Cytokines by Natural Killer (NK) Cells

**Figure 2:**
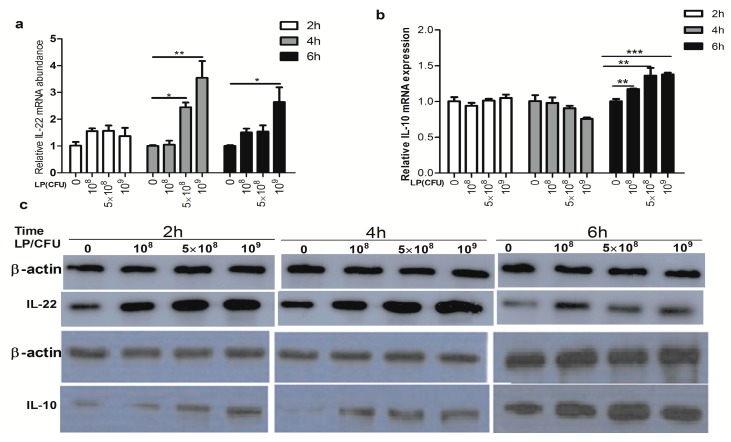

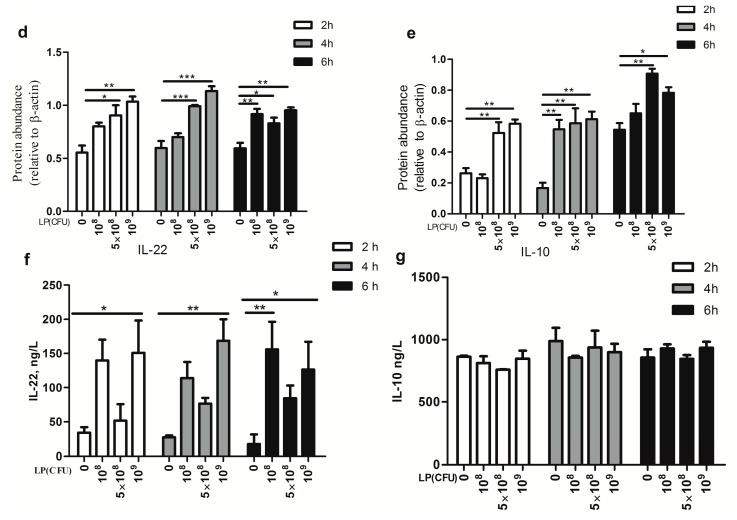
Effect of *Lactobacillus plantarum* (LP) on the expression of IL-22 and IL-10 in NK cells. NK cells were untreated or treated with LP (10^8^, 5 × 10^8^ or 10^9^ CFU/mL) for 2, 4, or 6 h. Cells were collected and the levels of IL-22 and IL-10 mRNA and protein were analyzed. (**a**,**b**) the relative transcript abundance of IL-10 and IL-22, respectively; (**c**) Western blot analysis of IL-22 and IL-10 protein. Loading and transfer of equal amounts of protein was confirmed by the immunodetection of β-actin; (**d**,**e**) the ratio of IL-22 and IL-10 to β-actin, respectively. Similar results were obtained from three independent experiments; (**f**,**g**) were the amount of IL-22 and IL-10 in media conditioned by NK cells, measured with IL-22 and IL-10-specific sandwich ELISAs. Data are means ± SEM. * *p* < 0.05, ** *p* < 0.01, *** *p* < 0.001.

There was no effect of LP on the IL-10 mRNA expression by NK cells at 2 and 4 h. Exposure to various concentrations (10^8^, 5 × 10^8^ and 10^9^ CFU/mL) of LP can significantly increase the mRNA expression of IL-10 at 6 h (Figure 2b). Higher concentrations (5 × 10^8^ and 10^9^ CFU/mL ) of LP markedly enhanced the abundance of IL-22 mRNA at 4 h, and only 10^9^ CFU/mL LP markedly increased the expression of IL-22 mRNA at 6 h (Figure 2a). The protein levels of IL-22 and IL-10, from Western blots, were significantly enhanced by various concentrations of LP at 2, 4, and 6 h (Figure 2c–e). However, the amounts of IL-22, but not IL-10, measured in the media by ELISA, were elevated by higher concentration of LP after 2, 4, and 6 h of culture (Figure 2f,g).

### 2.3. Lactobacillus plantarum Effectively Increased the Expression of Toll-Like Receptor 2 (TLR2)

**Figure 3:**
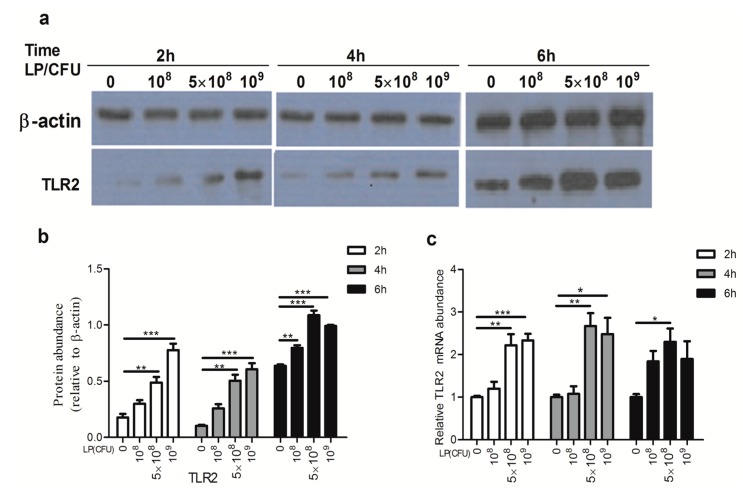
*Lactobacillus plantarum* (LP) increased the expression of TLR2. NK cells were untreated or treated with *L. plantarum* (10^8^, 5 × 10^8^ or 10^9^ CFU/mL) for 2, 4, or 6 h. Cells were collected and the level of TLR2 mRNA and protein was analyzed. (**a**) Western blots of the amounts of TLR2 protein. Loading and transfer of equal amounts of protein was confirmed by the immunodetection of β-actin; (**b**) The ratios of TLR2 to β-actin. Similar results were obtained from 3 independent experiments; (**c**) Relative expression of TLR2 mRNA. TLR2, Toll-like receptor 2. Data are means ± SEM. * *p* < 0.05, ** *p* < 0.01, *** *p* < 0.001.

As shown in Figure 3, concentrations of 5 × 10^8^ and 10^9^ CFU/mL of LP significantly increased the protein and mRNA expression of TLR2 at 2 and 4 h. After 6 h incubation, only 5 × 10^8^ CFU/mL of LP significantly up-regulated the TLR2 transcript, though the protein level of TLR2 was elevated by different experimental concentrations of LP.

### 2.4. The Effect of NK Cells Stimulated by Lactobacillus plantarum on the TEER of NCM460 Cells Challenged with Enterotoxigenic Escherichia coli (ETEC) K88

**Figure 4:**
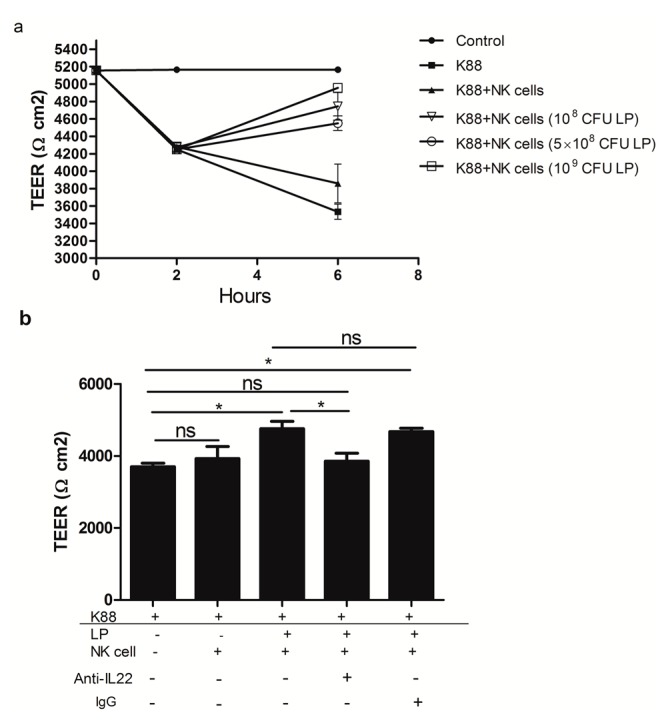
Changes in NCM460 cell monolayers Trans-Epithelial Electrical Resistance (TEER) after different treatments. (**a**) NCM460 cell monolayers were infected or not with 10^8^ CFU/mL of *enterotoxigenic Escherichia coli* (ETEC) K88 for 2 h, and then NK cells stimulated or not by *Lactobacillus plantarum* (LP) (10^8^, 5 × 10^8^, 10^9^ CFU/mL) were added to the NCM460 cell monolayers challenged with ETEC K88 and co-cultured for another 4 h. TEER values were determined at 0, 2, and 6 h post treatment; (**b**) NK cells stimulated by 10^9^ CFU/mL LP in the presence or absence of either 5.0 µg/mL polyclonal anti-IL-22 antibody against IL-22 or 5.0 µg/mL control goat IgG were added to the NCM460 cell monolayers challenged with ETEC K88. After the treatment, TEER was detected. Data are means ± SEM. * *p* < 0.05, ns: not significant. TEER, trans-epithelial electrical resistance.

As showed in Figure 4a, ETEC K88 infection significantly reduced NCM460 cell monolayers TEER. However, treatment of NCM460 cell monolayers with NK cells stimulated by 10^8^, 5 × 10^8^, 10^9^ CFU/mL LP significantly attenuated the decrease in TEER of NCM460 cells induced by ETEC K88. However, NK cells that were not primed with LP did not have an effect as the cells stimulated by LP on ETEC K88-induced drop in TEER. We further explore the relationship between the protecting role of IL-22 and the integrity of intestinal epithelial barrier. Importantly, we found that NK cells stimulated by 10^9^ CFU/mL LP in the presence of polyclonal anti-IL-22 antibody against IL-22, but not control goat IgG, led to significant decreases in TEER of NCM460 cell monolayers challenged with ETEC K88, when compared with cells stimulated by LP alone.

### 2.5. Lactobacillus plantarum-Treated NK Cells Increased Expression of Tight Junction Components in NCM460 Cells That Were Challenged with ETEC K88

**Figure 5:**
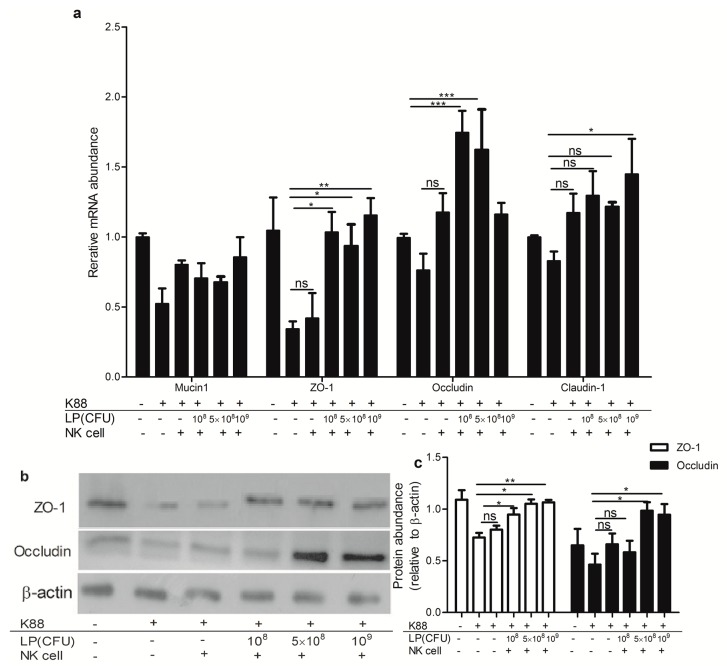
NK cells stimulated by *Lactobacillus plantarum* (LP) increased expression of tight junction proteins in NCM460 cells challenged with ETEC K88. NCM460 cell monolayers were untreated or treated with 10^8^ CFU/mL ETEC K88 for 2 h, then NK cells stimulated or not by LP (10^8^, 5 × 10^8^ or 10^9^ CFU/mL) were added to ETEC K88-infected NCM460 cells and co-cultured for another 4 h. NCM460 cells were collected and the expressions of tight junctions were analyzed. (**a**) Relative expression of mucin1, ZO-1, occludin and claudin-1 mRNA; (**b**) Western blots of the ZO-1 and occludin proteins. Equal protein loading was confirmed by analysis of β-actin in the protein extracts. (**c**) The ratios of ZO-1 and occludin to β-actin. Similar results were obtained from 3 independent experiments. Data are means ± SEM. * *p* < 0.05, ** *p* < 0.01, *** *p* < 0.001, ns: not significant. ZO-1, zonula occluden-1.

As shown in Figure 5, treatment of polarized NCM460 cell monolayer with ETEC K88 reduced relative expression of Mucin1, ZO-1, occludin and claudin-1 transcripts. However, adding NK cells stimulated by various doses (10^8^, 5 × 10^8^ and 10^9^ CFU/mL) of LP to ETEC K88-infected NCM460, led to a significant increase of relative abundance of ZO-1 transcript and protein level. NK cells stimulated by LP (5 × 10^8^ and 10^9^ CFU/mL) significantly increased the level of occludin mRNA and protein in ETEC K88-infected NCM460 cells. Furthermore, the NK cells stimulated by 10^9^ CFU/mL LP significantly increased the claudin-1 transcripts in ETEC K88-infected NCM460 cells. However, NK cells that were not primed with LP did not have the defensive effect as the cells stimulated by LP.

### 2.6. Lactobacillus plantarum-Treated NK Cells Up-Regulated the Expression of IL-22 Receptor

**Figure 6:**
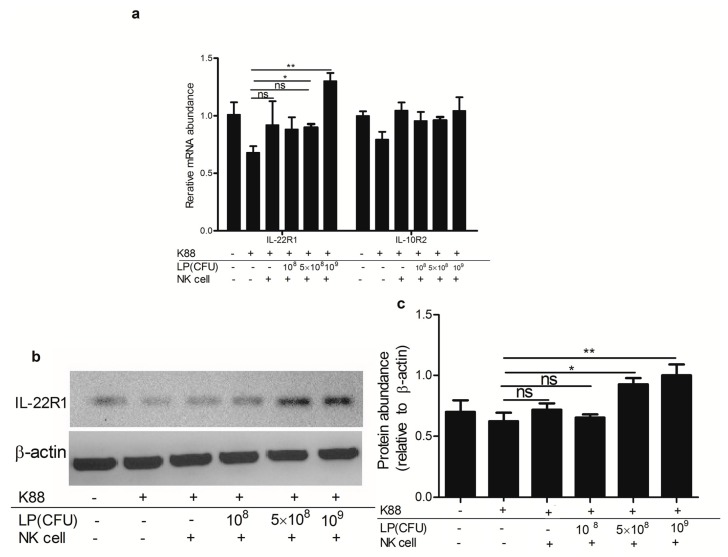
NK cells stimulated by *Lactobacillus plantarum* (LP) increased the expression of IL-22 receptor in ETEC K88-infected NCM460 cells. NCM460 cell monolayers were untreated or treated with 10^8^ CFU/mL ETEC K88 for 2 h, then NK cells stimulated or not by LP (10^8^, 5 × 10^8^ or 10^9^ CFU/mL) were added to ETEC K88-infected NCM460 cells and co-cultured for another 4 h. After treatments, NCM460 cells were collected and the expressions of IL-22R1 and IL-10R2 were analyzed. (**a**) The abundance of IL-22R1 and IL-10R2 mRNA; (**b**) The protein level of IL-22R1 was determined by Western blotting. Equal protein loading was confirmed by analysis of β-actin in the protein extracts; (**c**) The ratios of IL-22R1 to β-actin. Similar results were obtained from 3 independent experiments. Data are means ± SEM. * *p* < 0.05, ** *p* < 0.01, ns: not significant.

As shown in Figure 6, NK cells stimulated by 5 × 10^8^ and 10^9^ CFU/mL LP, significantly increased the expression of IL-22R1 mRNA without affecting that of IL-10R2 mRNA in ETEC K88-infected NCM460 cells. At the protein level, after incubation with NK cells stimulated by 5 × 10^8^ and 10^9^ CFU/mL LP, the protein level of IL-22R1 in ETEC K88-infected NCM460 cells was enhanced, in comparison to that in NCM460 cells infected with ETEC K88 alone. As compared with NK cells that were stimulated by higher concentrations LP, NK cells that were not primed with LP had no up-regulating effect on the expression of IL-22R1 or IL-10R2 in ETEC K88-infected NCM460 cells.

### 2.7. Lactobacillus plantarum-Treated NK Cells Activated Tyk2 and Stat3 in NCM460 Cells Challenged with ETEC K88

**Figure 7:**
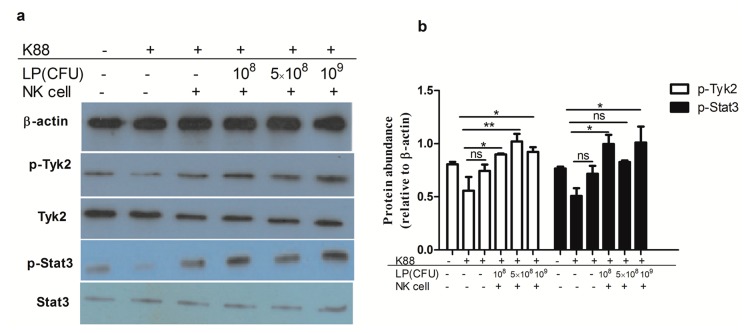
*Lactobacillus plantarum* (LP)-treated NK cells activated Tyk2 and Stat3 in NCM460 cells challenged with ETEC K88. NCM460 cell monolayers were untreated or treated with 10^8^ CFU/ml ETEC K88 for 2 h, then NK cells stimulated by LP (0, 10^8^, 5 × 10^8^ or 10^9^ CFU/mL) were added to ETEC K88-infected NCM460 cells and co-cultured for another 4 h. NCM460 cells were collected and protein abundances were analyzed. (**a**) Western blots showing the phosphorylation and total levels of Tyk2 and Stat3; (**b**) The ratios of p-Stat3 and p-Tyk2 to β-actin. Similar results were obtained from 3 independent experiments. Data are means ± SEM. * *p* < 0.05, ** *p* < 0.01, ns: not significant.

We next investigated the activation of Tyk2 and Stat3 in NCM460 cells that were exposed to ETEC alone or in ETEC-infected NCM460 cells that were subsequently co-cultured with NK cells stimulated by LP. Western blotting analysis showed that various concentrations of LP-treated NK cells induced phosphorylation of Tyk2 and Stat3 in NCM460 cells infected with ETEC K88, as compared with NCM460 cells that were exposed to ETEC alone (Figure 7).

## 3. Discussion

The present study was focused on addressing the interaction role of NK cells with LP that has a mucosal-protective function. It was therefore hypothesized that LP induced NK cells to exhibit an enhanced function that protected intestinal epithelial cell integrity against ETEC K88 infection. It was demonstrated here that LP effectively increased the protein levels of NCRs in NK cells. It has been shown that NCRs were highly and specifically expressed in NK cells [17,18]. These molecules can induce strong activation signals, which promote cytokine secretion by NK cells [19]. For example, IL-22 was preferentially produced by NCR1^+^ NK cells [14]. Observations have confirmed the existence of distinct NK cell subsets, not just as general pro-inflammatory killer cells [13,14]. Several studies have demonstrated a protective role for IL-22-producing NK cells in the regulation of gut inflammation [20]. In the present study, we first found that LP can drive IL-22 production in NK cells that might provide a protective effect against the damage to intestinal epithelial barrier by ETEC [13].

As a Gram-positive bacterium, the cell wall of LP contains lipoteichoic acid, and it lacks lipopolysaccharide (LPS). Here, LP was shown to significantly increase the expression in NK cells of TLR2, which plays a major role in recognition of Gram-positive bacteria [21]. Our result was consistent with the study by Rizzello who suggested that anti-inflammatory cytokines can be induced when microbes were recognized by TLR2 [22].

TEER is a direct indicator for evaluating epithelial permeability and integrity [23]. Our result was consistent with the study that ETEC K88 caused a marked reduction in monolayer TEER [2]. However, we found that NK cells stimulated by LP partially offset the decrease in NCM460 cell monolayer TEER caused by K88. Our study further showed that NK cells stimulated by 10^9^ CFU/mL LP in the presence of anti-human mAb specify against IL-22 cannot maintain TEER of NCM460 cell monolayers challenged with ETEC K88. Thus, we suggested that IL-22 significantly enhanced the recovery of epithelial resistance and that IL-22 was crucial for maintaining barrier function at epithelial surfaces [24].

A previous study has reported that IL-22-expressing NK cells mediated protection from IBD-induced gut injury [25]. A study by Tsai et al. demonstrated that IL-22-induced claudin-2 up-regulation drove diarrhea and pathogen clearance [26]. In ETEC K88-infected NCM460 cells, decreased tight junction proteins expression of ZO-1, claudin-1, and occludin, which are known to play important roles in intestinal epithelial barrier function [27], were largely prevented by NK cells stimulated by LP, but not NK cells that were not primed with LP, suggesting that NK cells stimulated by LP provided a protective effect against the ETEC-induced injury to the integrity of intestinal epithelial barrier by maintaining the expression of tight junction proteins.

IL-22 exerts its biological effects via a receptor complex consisting of the receptor chains IL-22R1 and IL-10R2, which are expressed in epithelial cells of the small and large bowel [28,29]. IL-10R2 also is an accessory receptor chain for IL-10, while signaling through IL-22R1 is confined to IL-22 [30]. A previous study has shown that intestinal epithelial IL-22R1 signaling contributed to host defense during *C. rodentium* infection and that intestinal epithelial IL-22R1 knockout resulted in the reduction of mucosal IL-22 production [31]. Consistent with this result, the data presented here shows that the stimulation of ETEC-infected NCM460 cells with LP-treated NK cells significantly increased the transcripts of IL-22R1 and its protein without affecting the expression of IL-10R2. Hence, IL-22 produced by LP-treated NK cells seemed to play a preferred protective role in epithelial cells against ETEC K88 challenge. Our result was consistent with the study that has identified a critical function of the IL-22-IL-22R1 pathway in the protection of the integrity of barrier function [32].

IL-22 binding to its receptor complex could induce activation of Stat3 and Tyk2 pathways [33]. Consistent with those results, it was found here that LP-treated NK cells increased the phosphorylation of Stat3 and Tyk2 in ETEC K88-infected NCM460 cells. Pickert et al. demonstrated that intestinal epithelial Stat3 activation regulated immune homeostasis in the gut by promoting IL-22-dependent mucosal wound healing [34]. The present results provide a molecular explanation for NK cells stimulated by LP conferring protection of intestinal epithelial NCM460 cells against ETEC K88 induced damage, probably by activation of Stat3 and Tyk2 signaling.

Collectively, it is demonstrated here that LP enhanced IL-22 production in NK cells that might be able to protect the integrity of intestinal epithelial barrier despite infection with ETEC K88.

## 4. Materials and Methods

### 4.1. Cell Culture

NK-92MI cells were obtained from the American Type Culture Collection (Manassas, VA, USA). NK-92MI cells were maintained in the alpha modification of Eagle’s minimum essential medium (α-MEM; Invitrogen, Carlsbad, CA, USA) containing 2 mm l-glutamine, 0.2 mm inositol, 0.02 mm folic acid, 0.01 mm 2-mercaptoethanol (Sigma-Aldrich Corporation, St. Louis, MO, USA), 12.5% FBS and 12.5% horse serum (Invitrogen). The NK-92MI cells used in this study were at a stage of 5 to 18 passages. NCM460 cells derived from normal human colon mucosal epithelium, have been used widely in intestinal research [35]. This cell line was kindly supplied by Xingfeng Gao, Guangdong Provincial People’s Hospital, and were used here at a stage of 10 to 18 passages. Cells were cultured in uncoated plastic culture flasks (100 mm^2^) in DMEM-F12 containing 10% fatal bovine serum (FBS) at 37 °C under 5% CO_2_. Culture medium was changed every 2 day and cells were passaged by trypsinization at 80–90% confluency.

### 4.2. Bacterial Growth

*L. plantarum* strain CGMCC1258 (kindly provided by Hang Xiaomin, Institute of Science Life, Shanghai Jiao Tong University, Shanghai, China), was originally isolated from the feces of healthy infants [36]. LP was grown in Man Rogosa Sharp (MRS) medium at 37 °C under anaerobic conditions. After overnight incubation, bacteria were centrifuged at 5000× *g* for 10 min at 4 °C, washed with cold phosphate buffered saline (PBS), and then resuspended in antibiotic-free α-MEM medium.

ETEC K88 strain (serotype O149: K91: K88ac) was grown in Luria-Bertani (LB) medium at 37 °C with vigorous shaking. After overnight incubation, ETEC K88 were then collected by centrifugation, washed with cold phosphate buffered saline (PBS), and then resuspended in antibiotic-free DMEM-F12 medium.

### 4.3. Treatment of NK Cells with L. plantarum

NK-92MI cells (10^6^ cells/well) were seeded into 6-well Transwell inserts (24 mm, 4.67 cm^2^, Corning, Amsterdam, The Netherlands). After 24 h incubation, the medium in the basolateral side was removed and replaced with 2.6 mL α-MEM (without streptomycin/penicillin), containing different concentrations of LP (10^8^, 5 × 10^8^ and 10^9^ CFU/mL). Control NK cells were cultured only with α-MEM. The NK cells and LP were indirectly incubated for 2, 4, or 6 h at 37 °C under 5% CO_2_. After that, conditioned media from the apical compartments were collected for subsequent assay of IL-22 and IL-10 (4 replicates), and the NK cells from the apical compartment were collected for subsequent real-time quantitative PCR (qPCR) or Western blot analysis.

Subsequently, we chose 4 h incubation for further experimentation. NK cells were untreated or treated with different concentrations of LP (10^8^, 5 × 10^8^ and 10^9^ CFU/mL) for 4 h, and then the NK cells and the culture medium from the apical compartment were collected for stimulating ETEC K88-infected NCM460 cells.

### 4.4. The Effect of NK Cells Stimulated by L. plantarum on the Function of ETEC K88-Infected NCM460 Cells

NCM460 cells (3.7 × 10^5^) were seeded into 6-well Transwell inserts (24 mm, 4.67 cm^2^, Corning, Amsterdam, The Netherlands) and until their trans-epithelial electrical resistance was stable (about 5000 Ωcm^2^). The monolayer of NCM460 cells were untreated or treated with 1.5 mL medium containing 10^8^ CFU/mL ETEC K88 (without streptomycin/penicillin) for 2 h at 37 °C under 5% CO_2_, followed by the removal of the non-attached bacteria and three gently washes with PBS. Then, ETEC-infected NCM460 cells were co-cultured with NK cells that were stimulated or not by LP (10^8^, 5 × 10^8^ and 10^9^ CFU/mL) and their conditioned medium in the apical compartments for another 4 h. After removing the suspended NK cells, NCM460 cells were washed twice with PBS, and then, collected and stored at −80 °C until further analysis by Western blotting or qPCR. NCM460 cells were cultured only with DMEM-F12 medium as the control group; NCM460 cells treated with ETEC K88 only, NCM460 cells pre-treated with ETEC K88, and then exposed to NK cells that were not primed with LP, and NCM460 cells pre-treated with ETEC K88 and then exposed to NK cells stimulated by 10^8^, 5 × 10^8^ or 10^9^ CFU/mL LP were the treated groups.

### 4.5. Measurement of Trans-Epithelial Electrical Resistance (TEER)

NCM460 cells (3.7 × 10^5^) were seeded into 6-well Transwell inserts (24 mm, 4.67 cm^2^, Corning, Amsterdam, The Netherlands) and TEER was measured every day using Millicell ERS-2 Voltohmmeter^®^ (Millipore, Billerica, MA, USA) until the value reached stable (about 5000 Ωcm^2^). ETEC K88 were diluted in DMEM-F12 without streptomycin/penicillin and added to the apical compartment for 2 h treatment. Control NCM460 cells were cultured only with DMEM-F12 medium. The TEER measurement was determined at 2 h after the treatment. Followed by the gentle removal of the non-attached bacteria, ETEC-infected NCM460 cells were co-cultured with NK cells stimulated by different concentrations of LP in the apical compartments for another 4 h. After the treatment, the TEER assay was performed again. TEER (Ωcm^2^) = (Total resistance − Blank resistance) (Ω) × Area (cm^2^).

### 4.6. The Effect of IL-22 on Trans-Epithelial Electrical Resistance (TEER) of NCM460 Cell Monolayer Challenged with ETEC-K88

NK-92MI cells (10^6^ cells/well) were seeded into 6-well Transwell inserts (24 mm, 4.67 cm^2^, Corning). After 24 h incubation, the medium in the basolateral side was removed and replaced with 2.6 mL α-MEM (without streptomycin/penicillin) containing 10^9^ CFU/mL LP. At the same time, 5.0 μg/mL of polyclonal anti-IL-22 antibody against IL-22 (Cat number: AF782, purchased from R&D, Minneapolis, MN, USA, and were obtained from Zhongshan School of Medicine, Sun Yat-sen University) or 5.0 µg/mL control goat IgG (purchased from R&D) was added to the apical compartments. Control NK cells were cultured only with α-MEM. NK cells were treated with 10^9^ CFU/mL LP in the presence or absence of either polyclonal anti-IL-22 antibody against IL-22 or control goat IgG for 4 h. After that, conditioned media from the apical compartments and the NK cells were collected and added to the ETEC-infected NCM460 cell monolayers and co-cultured for another 4 h. After the treatment, the TEER assay was detected.

### 4.7. Enzyme-Linked Immunosorbent Assay (ELISA)

The concentrations of IL-22 and IL-10 proteins in media that was conditioned by the culture of NK cells were determined using ELISA kits (Raybiotech, Norcross, GA, USA), following the manufacturer’s instructions.

### 4.8. Real-Time Quantitative PCR (QPCR) Analysis

QPCR was done as we previously described [37]. Briefly, cells were harvested and total RNA was isolated with Trizol Reagent (Invitrogen). Quality and integrity of RNA was assessed on 1.0% agarose gel electrophoresis with visualization of complete 28S and 18S bands. Purity and concentration of RNA were measured by a NanoDrop-ND1000 spectrophotometer (Thermo Fisher Scientific Inc., Walldorf, Germany) with A260/280 of 1.91–1.98 and A230/260 of 2.00–2.15. 1 µg of total RNA was reverse transcripted into cDNAs using PrimeScript™II 1st Strand cDNA Synthesis Kit (Takara, Tokyo, Japan) according to the manufacturer’s guidelines. qPCR amplification was performed with SYBR green I (Takara), 10-fold diluted cDNA and gene-specific primers (Table 1). Parameters for qPCR were 95 °C × 3 min, followed by 40 cycles of 95 °C × 15 s, 60 °C × 30 s, 72 °C × 30 s. All of the samples were amplified in triplicate. The β-actin gene was used as an internal control because its transcript expression was not markedly influenced by any treatment in the present study (results not shown). The mRNA expression of the target genes, relative to β-actin was determined using 2^−ΔΔ*C*t^ method, Δ*C*_t_ = *C*_t (target gene)_ − *C*_t (β-actin)_ and ΔΔ*C_t_* = Δ*C*_t (Treatment)_ − Δ*C*_t (Control)_.

### 4.9. Western Blotting

Cells were rapidly washed twice with ice-cold PBS and then lysed for 30 min at 4 °C in 0.3 mL of RIPA buffer consisting of 1% Triton X-100, 0.5% Nonidet P-40, 150 mm NaCl, 10 mm Tris HCl (pH 8.0), 1 mm EDTA, 1 mm EGTA, 0.2 mm Na_3_VO_4_, 0.2 mm phenylmethylsulfonyl fluoride, 50 mm NaF, 30 mm Na_4_P_2_O_7_, 1% protease inhibitor cocktail and 1% phosphatase inhibitor. The cell lysates were centrifuged (13,000× *g* for 15 min at 4 °C). A BCA protein assay kit (Pierce, Rockford, IL, USA) was used to assay protein concentration in the supernatant fluid, and bovine serum albumin was used as a standard. Protein samples were adjusted to an equal amount and were diluted with 5× loading buffer, heated at 100 °C for 10 min, cooled on ice, then used for Western blot analysis. 8–12% SDS-PAGE was used to separate denatured proteins, and then separated denatured proteins were transferred to nitrocellulose membranes. After blocking with 3% BSA in TBST buffer for 10 min, membranes were washed three times and were incubated with the diluted primary antibodies overnight at 4 °C. Subsequently, the three times washed membranes were then incubated with the appropriate HRP-labeled secondary antibodies for 1 h at room temperature. After three 10 min washes, chemiluminescent HRP substrate (Millipore, Billerica, MA, USA) and a VersaDoc imaging system (Bio-Rad, California, CA, USA) were used to detect immunoreactive proteins. The band intensity was quantified using Image J software (National Institutes of Health, Bethesda, MD, USA). Primary antibodies for β-actin (Cat number: #4970), TLR2 (Cat number: #12276), Tyk2 (Cat number: #14193), p-Tyk2 (Cat number: #68790), Stat3 (Cat number: #4904) and p-Stat3 (Cat number: #9145) (these antibodies were purchased from Cell Signaling Technology, Boston, MA, USA); IL-22 (Cat number: ab5984), IL-10 (Cat number: ab34843), IL-22R1 (Cat number: ab5984), NCR1 (Cat number: ab199128), NCR2 (Cat number: ab133668), NCR3 (Cat number: ab186425), zonula occluden-1 (ZO-1) (Cat number: ab96587) and occludin (Cat number: ab167161) (These antibodies were purchased Abcam, Hong Kong, China) were used in this study. The results are expressed as the abundance of each target protein relative to β-actin.

### 4.10. Statistical Analysis

Effects of the treatment were evaluated by one-way ANOVA. Data are presented as means ± SEM. Analysis was performed using GraphPad Prism Version 5 (GraphPad Software, La Jolla, CA, USA). A value of *p* < 0.05 was considered to be statistically significant.

## Figures and Tables

**Table 1:** Primer Sequences Used in this Study.

Genes	Sequences (5′-3′)		Product Size (bp)	% Primer Efficiency (E)	GenBank Accession
*IL-10*	Forward	AGAGGTCTCCAAAATCGG	160	95.4	NM_000572.2
	Reverse	GGCTTCTTTCTAAATCGTTC			
*IL-22*	Forward	AGTGCTGTTCCCTCAATCT	203	94.8	NM_020525.4
	Reverse	GCAAATCCAGTTCTCCAAT			
*TLR2*	Forward	CTCTACCAGATGCCTCCCT	128	102.2	NM_001318787.1
	Reverse	ATTGCCACCAGCTTCCA			
*IL-10R2*	Forward	AGGGCTGAATTTGCAGATGA	213	98.8	NM_000628.4
	Reverse	CCGTTTTTCCAGTATTGCAC			
*IL-22R1*	Forward	CCCCACTGGGACACTTTCTA	243	96.9	NM_021258.3
	Reverse	TGGCCCTTTAGGTACTGTGG			
*ZO-1*	Forward	CAACATACAGTGACGCTTCACA	105	105.5	NM_001301025.2
	Reverse	CACTATTGACGTTTCCCCACTC			
*Occludin*	Forward	ACAAGCGGTTTTATCCAGAGTC	89	98.3	NM_001205254.1
	Reverse	GTCATCCACAGGCGAAGTTAAT			
*Claudin1*	Forward	AAATCAGAACTTTGGAGGC	103	97.9	NM_021101.4
	Reverse	AAACAAGAGTGCTATGGGTC			
*Mucin1*	Forward	CGCCTGCCTGAATCTGTT	281	104.7	NM_001018016.2
	Reverse	GCTCTTGGTAGTAGTCGGTGC			
*β-actin*	Forward	TAGTTGCGTTACACCCTTTC	153	99.9	NM_001101.3
	Reverse	TGTCACCTTCACCGTTCC			
